# The Sonodynamic Effect of Curcumin on THP-1 Cell-Derived Macrophages

**DOI:** 10.1155/2013/737264

**Published:** 2012-12-30

**Authors:** Fengping Wang, Qianping Gao, Shuyuan Guo, Jiali Cheng, Xin Sun, Qiannan Li, Tengyu Wang, Zhiguo Zhang, Wenwu Cao, Ye Tian

**Affiliations:** ^1^Department of Cardiology, The First Affiliated Hospital of Harbin Medical University, Harbin 150001, China; ^2^Department of Emergency, The Second Affiliated Hospital of Harbin Medical University, Harbin 150086, China; ^3^Department of Physics, Harbin Institute of Technology, Harbin 150080, China; ^4^Materials Research Institute, The Pennsylvania State University, University Park, State College, PA 16802, USA; ^5^Department of Pathophysiology, Harbin Medical University, Harbin 150086, China

## Abstract

Curcumin is extracted from the rhizomes of the traditional Chinese herb *Curcuma longa* and has been proposed to function as a photosensitizer. The potential use of curcumin as a sonosensitizer for sonodynamic therapy (SDT) requires further exploration. This study investigated the sonodynamic effect of curcumin on macrophages, the pivotal inflammatory cells in atherosclerotic plaque. THP-1-derived macrophages were incubated with curcumin at a concentration of 40.7 **μ**mol/L for 2 h and then exposed to pulse ultrasound irradiation (2 W/cm^2^ with 0.86 MHz) for 5–15 min. Six hours later, cell viability was decreased in cells that had been treated with ultrasound for 10 and 15 min. After ultrasound irradiation for 15 min, the ratio of apoptotic and necrotic cells in SDT group was higher than that in ultrasound group, and the ratio of apoptotic cells was higher than that of necrotic cells. Both loss of mitochondrial membrane potential and morphological changes of cytoskeleton were apparent 2 h after treatment with curcumin SDT. These findings support that curcumin had sonodynamic effect on THP-1-derived macrophages and that curcumin SDT could be a promising treatment for atherosclerosis.

## 1. Introduction 

Atherosclerosis poses a severe threat to human health. Most acute cardiovascular events result from the rupture of an atherosclerotic plaque, and macrophages play a crucial role in the progression [1–3]. Decreasing the infiltration of an atherosclerotic plaque by macrophages could stabilize the plaque and inhibit its progression. Photodynamic therapy (PDT) for atherosclerosis is a new treatment modality that has been proven to induce plaque regression in animal atherosclerosis models [4, 5]. The mechanism may involve macrophage apoptosis induced by PDT [6]. However, PDT has two recognized drawbacks: (i) it can only be applied to superficial lesions because of the limited penetration of light into tissues, even though atherosclerotic lesions may exist deep in the human body. (ii) PDT-treated patients tend to suffer long-lasting skin sensitivity due to the retention of photosensitizers in skin, and they may need to spend several weeks in the dark after such treatment.

To resolve the problem of tissue penetration, another method called sonodynamic therapy (SDT) has been investigated. Ultrasound has an appropriate tissue attenuation coefficient, allowing it to penetrate into tissues and reach nonsuperficial objects while maintaining the ability to focus energy into small volumes and activate sonosensitizers. Among noninvasive treatment options, this advantage is unique compared to the use of laser light for photodynamic therapy. The basis of the therapy is to administer a small amount of sonosensitizer, which is selectively taken up by target cells, and then expose the target lesion to ultrasound to activate sonosensitizer [7]. Until now, there have been extensive investigations of the effects of SDT on tumors [8]. However, this technique has not been applied to atherosclerosis.

The sonosensitizer is crucial during SDT in order to enhance the cytotoxicity of the ultrasound. Photochemically active hematoporphyrin derivatives (HPDs), including hematoporphyrin, photofrin II, ATX-70, and ATXS10, have been demonstrated to induce cell death when activated by ultrasound irradiation, indicating that these chemicals, which were originally generated for PDT, are applicable as sonosensitizers [9]. However, HPDs are likely to cause photodermatitis and are not generally used in clinical practice. To avoid photodermatitis, development of a new sonosensitizer that can be widely used is necessary.

Curcumin is the major constituent of *turmeric* powder, which is extracted from the rhizomes of the plant *Curcuma longa*. As a powder, *turmeric* is widely used as a coloring and flavoring spice in foods as well as in folk medicine for the management of various inflammatory disorders and wound healing [10]. Curcumin has been described as having antioxidant, anti-inflammatory, and anticarcinogenic properties [11–13]. It can also protect against lipid-induced damage in the inflammatory cells of the vascular system by the upregulation of FOXO3a activity [14]. To our knowledge, there has been no report of photodermatitis caused by curcumin. In recent years, curcumin derivatives have been demonstrated to reduce aortic fatty streak formation and to protect animal models against atherosclerosis [15–17]. It has been reported that the use of curcumin as a photosensitizer has induced apoptosis of tumor cells through activation of caspase pathways [18]. However, whether curcumin could be used as a sonosensitizer is still unknown. 

We hypothesized that curcumin would have a sonodynamic effect on macrophages, which would enable curcumin SDT to potentially be used as a treatment for atherosclerosis. In this study, we used curcumin to mediate the effects of SDT on macrophages in order to determine whether it can induce cell apoptosis.

## 2. Materials and Methods 

### 2.1. Chemicals

Curcumin was provided by the National Institute for the Control of Pharmaceutical and Biological Products (Beijing, China). The reagent was a commercial product of analytical grade with purity ≥ 98%. It was dissolved in 100% dimethyl sulfoxide (DMSO) (Sigma, USA) and stored at −20°C in dark. The stock solutions were diluted 10- to 10^3^-fold in the final experimental conditions. The final concentration of DMSO in cells was 0.1%. RPMI Medium (1640) and bovine fetal serum were obtained from HyClone Chemical Co. (HyClone, Logan, UT, USA). Phorbol-12-myristate-13-acetate (PMA) was obtained from EMD Biosciences, Inc. (La Jolla, USA). Hoechst 33342 and propidium iodide (PI) were from Sigma. JC-1 (5,5′,6,6′-Tetrachloro-1,1′,3,3′-tetraethyl-imidacarbocyanine iodide) probe was purchased from Beyotime (China). A rabbit polyclonal antibody against vimentin (ab8545) was purchased from Abcam (Hong Kong) Ltd. A rabbit polyclonal antibody against *β*-tubulin (H-235) and a goat polyclonal antibody against  *α*-actin (C-11) were purchased from Santa Cruz Biotechnology, Inc (USA). All other reagents were obtained from Sigma Chemical Co. Ltd.

### 2.2. Measurement of Absorption and Fluorescence Spectra of Curcumin

The absorption spectrum of curcumin was measured with a spectrophotometer (USB 2000, Ocean Optics Incorporated, FL, USA) under a 40 W wolfram lamp. The fluorescence spectrum of curcumin was measured with a spectrophotometer at a wavelength of 405 nm.

### 2.3. Cell Culture

Human THP-1 cells [19] (ATCC, USA) were cultured in RPMI Medium (1640) supplemented with fetal bovine serum (FBS) and Penicillin-Streptomycin (56.1 **μ**mol/L penicillin and 27.4 **μ**mol/L streptomycin). The cells were maintained at 37°C, with 5% CO_2_/95% air in a humidified incubator, and they were harvested for passage when they reached confluence. For experiments, cells were plated into microculture plates at 1 × 10^5^ cells/mL (Costar, Corning Incorporated, USA) in their usual medium plus PMA at a concentration of 16.2 **μ**mol/L (a final concentration of 162 nmol/L) for 72 h. Then the medium was removed and replaced with fresh medium without PMA.

### 2.4. Detection of Intracellular Uptake of Curcumin

Cells were grown to confluence in 24-well culture plates in standard culture conditions. Curcumin was added at a final concentration of 13.6–81.4 **μ**mol/L to the wells, which had been seeded with 1 × 10^4^ cells/mL. After 2 h, the cells were washed twice with PBS, and the curcumin that was taken up by the cells was examined by a fluorescence microscope (IX71, OLYMPUS, Japan) using a filter with an excitation wavelength of 420–480 nm and an emission wavelength of 480–550 nm. 

### 2.5. MTT Assay after SDT

The cells were seeded into flat plates with a diameter of 3.5 cm and incubated with 40.7 **μ**mol/L curcumin for 2 h in the dark. They were then exposed to pulse ultrasound (Sheng Xiang Technology, 838A-H-O-S multifunctional ultrasonic therapeutic device, China) at a power of 2 W/cm^2^ with 0.86 MHz for 5–15 min [20]. Control plates were sham-exposed to ultrasound. After SDT, each flat plate was incubated for 6 h. The survival rate of the cells was measured by MTT assay. All experiments were repeated three times independently.

### 2.6. Hoechst/PI Staining after SDT

The cells were divided into four groups including control (cells alone), curcumin treated (40.7 **μ**mol/L), ultrasound-treated (2 W/cm^2^ for 15 min), and SDT (ultrasound 2 W/cm^2^ for 15 min and curcumin 40.7 **μ**mol/L). Six hours after SDT, the cells were stained with Hoechst 33342 at 8.1 **μ**mol/L for 5 min, and they were then stained with PI at 15.0 **μ**mol/L for 5 min. The cell monolayer was washed twice with PBS and then examined under a fluorescence microscope with an excitation wavelength of 330–385 nm and emission wavelength of 420–480 nm. The percentages of apoptotic and necrotic cells were calculated from the total cell numbers. All cells from ten random microscopic fields at 40x magnification were counted. Experiments were repeated three times independently.

### 2.7. Cytoskeletal Protein Immunofluorescent Staining after SDT

Two hours after SDT, the cells were fixed with paraformaldehyde (PFA). Then the cells were perforated with a detergent such as Triton X-100 to allow exposure of the antibodies to the structures inside the cells. To avoid nonspecific binding of the second antibody, the cells were blocked with 1% BSA at room temperature for 1 h. Primary antibodies without fluorophore were added at 37°C for 1 h. Then the secondary antibody, which was conjugated with FITC (fluorescein isothiocyanate), was added at 37°C for 2 h. DAPI was added at room temperature for 2 min. The cell monolayer was washed twice with PBS and then examined under a fluorescence microscope with an excitation wavelength of 330–385 nm and emission wavelength of 420–480 nm, as well as an excitation wavelength of 420–480 nm and an emission wavelength of 480–550 nm. The status of cytoskeletal protein polymerization was quantitated by randomly choosing 10 microscopic fields at 40x magnification and counting cells in the field as either having cytoskeletal filaments that were intact or disturbed. The proportion of cells with disturbed cytoskeletal filaments was expressed as “the number of cells with disturbed cytoskeletal filaments/the total number of cells.”

### 2.8. Mitochondrial Membrane Potential (MMP,  Δ*ψm*) Assay after SDT

The loss of mitochondrial membrane potential (Δ*ψm*) was quantitatively determined by flow cytometry using the lipophilic cationic probe JC-1. When the cell is in a normal state, MMP is high and JC-1 predominantly appears as red fluorescence. When the cell is in an apoptotic or necrotic state, the MMP is reduced, and JC-1 appears as a monomer indicated by green fluorescence [21]. A change in the florescence from red to green indicates a decrease in the MMP. Tow hours after SDT, the cells were then washed with PBS and incubated with JC-1 working solution for 20 min at 37°C in the dark. Cells were washed with PBS and resuspended in 500 *μ*L PBS. The stained cells were analyzed by flow cytometry to determine the change in the florescence from red to green.

### 2.9. Statistical Analysis

All values are expressed as the means ± standard deviation. The Dunnett-T and SNK tests were used to assess the effects of varying curcumin concentration without irradiation on cell viability. The LSD and SNK tests were used to assess the effects of sonoactivated curcumin on cell viability.  *P*  value < 0.05 was considered to be significant.

## 3. Results

### 3.1. Intracellular Accumulation of Curcumin

**Figure 1:**
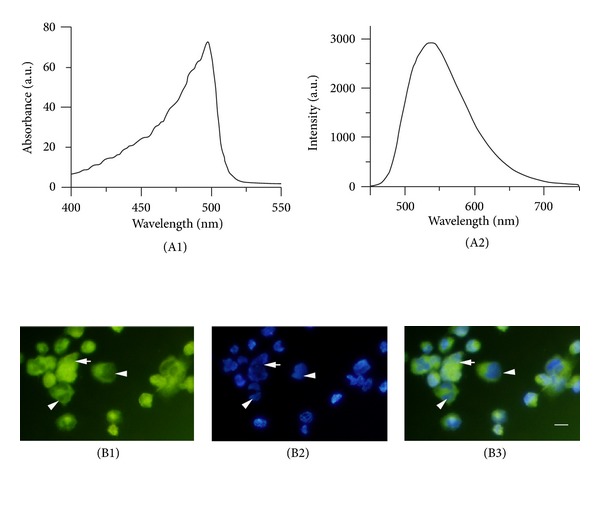
The spectrum of curcumin and the intracellular accumulation of curcumin (×400). Panel A1 is the absorption spectrum of curcumin under a wolfram lamp. panel A2 is the fluorescence emission spectrum of curcumin at 405 nm. Panel B1 is curcumin fluorescence in unfixed macrophages. Panel B2 is nuclei of the same cells after staining with Hoechst 33342. Panel B3 is a merged image of B1 and B2. Scale bar: 20 **μ**m. The dye appeared to be distributed throughout the cells (arrows), and in some cells, it was distributed in the cytoplasm only (arrowhead).

As shown in Figure 1, the absorption wavelength of curcumin was less than 520 nm, and the fluorescence emission wavelengths of curcumin ranged from 470 nm to 700 nm. The dye appeared to be distributed throughout cells, and in some cells, it was distributed in the cytoplasm only.

### 3.2. Cell Viability after Curcumin SDT

**Figure 2:**
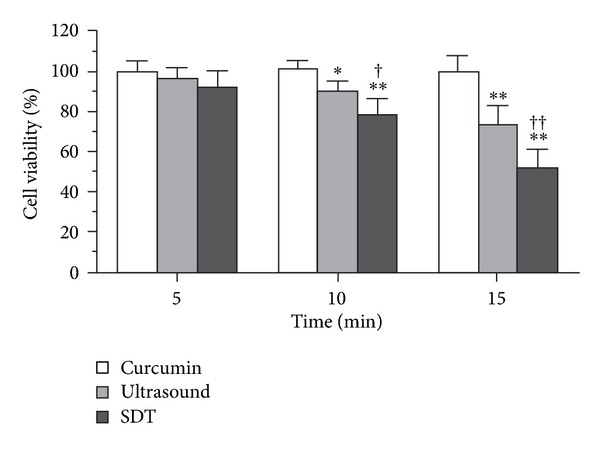
Cell viability after curcumin SDT. The effect of curcumin SDT on cell viability was assessed by MTT assay. Our results showed that the cell viability decreased with increasing time of ultrasound exposure. ^†^ *P* < 0.05, ^††^ *P* < 0.01 versus ultrasound alone; *P < 0.05, **P < 0.01 versus control.

As shown in Figure 2, curcumin SDT decreased cell viability more significantly [from 78.46 ± 8.22% (10 min) to 51.69 ± 9.39% (15 min)] than treatment with ultrasound alone [from 90.50 ± 4.74% (10 min) to 73.51 ± 9.42% (15 min)]. Cell viability was not significantly affected in cells treated for 5 min (*P* > 0.05). Treatment with curcumin alone did not affect cell viability compared to control (*P* > 0.05). DMSO at a concentration of 0.1% showed no effect on cell viability after ultrasound irradiation (data not shown). 

### 3.3. The Apoptosis and Necrosis of Cells 

**Figure 3:**
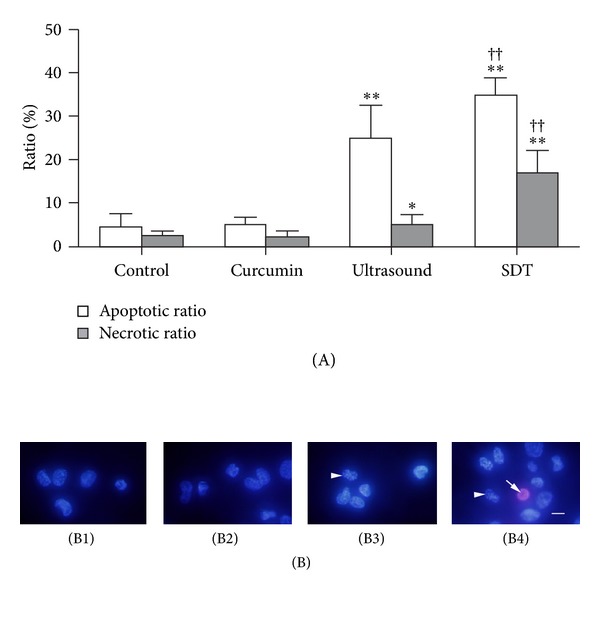
Apoptosis and necrosis of macrophages induced by curcumin SDT (×400). Panel A is the ratio of apoptotic and necrotic macrophages 6 h after curcumin SDT. *P < 0.05; **P < 0.01 versus control; ^††^ *P* < 0.01 versus ultrasound alone. B1 is control; B2 is curcumin alone; B3 is ultrasound irradiation alone; B4 is curcumin SDT. Scale bar: 20 **μ**m. Control and curcumin-treated cells showed uniform blue fluorescence in B1 and B2. Apoptotic cells were seen as bright blue fluorescent spots and are shown in B3 and B4 (arrowheads). Necrotic nuclei showed pink fluorescence in B4 (arrow).

As shown in Figure 3(A), the ratio of apoptotic cells in the SDT and ultrasound-treated groups was higher than that of control (34.90 ± 4.01% versus 4.41% ± 2.98%, *P* < 0.01; 25.02 ±7.45% versus 4.41% ± 2.98%, *P* < 0.01). The ratio of apoptotic cells in the SDT group was higher than that in the ultrasound group (34.90 ± 4.01% versus 25.02 ± 7.45%, *P* < 0.01). The ratio of necrotic cells in the SDT group and the ultrasound group was higher than that of control (16.91 ± 5.01% versus 2.26% ± 1.10%, *P* < 0.01; 4.97 ± 2.31% versus 2.26% ± 1.10%, *P* < 0.05). The ratio of necrotic cells in the SDT group was higher than that in the ultrasound group (16.91 ± 5.01% versus 4.97 ± 2.31%, *P* < 0.01). There was no difference apoptotic and necrotic cells between the curcumin-treated group and the controls. As shown in Figure 3(B), normal cells showed uniform blue fluorescence; apoptotic cells were seen as bright blue fluorescence spots, and necrotic nuclei were identified by the presence of staining with PI, which was evident as pink fluorescence.

### 3.4. Morphological Changes of the Cytoskeleton

**Figure 4:**
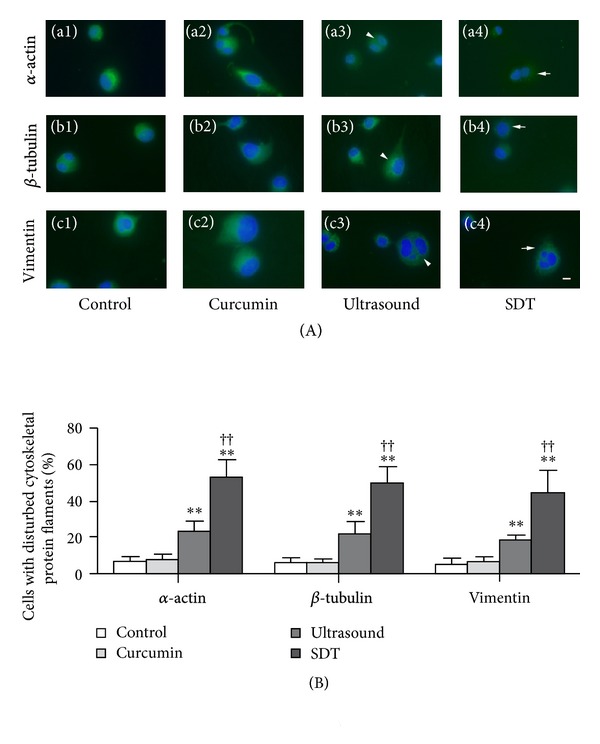
Changes in the cytoskeleton induced by curcumin SDT (×400). (A)-a1, b1, and c1 and (A)-a2, b2, and c2 are control and curcumin-treated cells showed a regular cytoskeletal network. (A)-a3, b3, and c3 are the fluorescence signal of cytoskeletal protein was slightly attenuated, as shown by arrowhead. (A)-a4, b4, and c4 are in some cells, cytoskeletal protein filaments diffused obviously, formed clusters and the plasma membrane lost its normal structure, as shown by arrow. Scale bar: 20 **μ**m. (B) is the percentage of cells with disturbed cytoskeletal filaments in each group. **P < 0.01 versus control, ^††^ *P* < 0.01 versus ultrasound group.

As shown in Figure 4(A), control cells showed a regular cytoskeletal network (green fluorescence), and the nucleus showed uniform blue fluorescence. The fluorescence signal of the cytoskeletal protein was slightly attenuated 2 h after treatment in some cells, as shown in Figure 4(A)-a3, b3, and c3. In the case of cells treated with curcumin SDT,  *α*-actin, *β*-tubulin, and vimentin filaments diffused obviously, formed clusters, and the plasma membrane lost its normal structure shown in Figure 4(A)-a4, b4, and c4. As shown in Figure 4(B), the percentage of cells with disturbed cytoskeletal filaments in the SDT group and the ultrasound group was higher than that in the control group (*α*-actin: 53.41 ± 9.48% versus 6.72% ± 2.54%, *P* < 0.01; 23.42 ± 5.43% versus 6.72% ± 2.54%, *P* < 0.01. *β*-tubulin: 49.89 ± 9.13% versus 6.06 ± 2.61%, *P* < 0.01; 21.94 ± 6.72% versus 6.06 ± 2.61%, *P* < 0.01. Vimentin: 44.48 ± 12.48% versus 4.95% ± 3.44%, *P* < 0.01; 18.83 ± 2.25% versus 4.95% ± 3.44%, *P* < 0.01). The percentage of cells with disturbed cytoskeletal filaments in the SDT group was higher than that in ultrasound group (*α*-actin: 53.41 ± 9.48% versus 23.42 ± 5.43%, *P* < 0.01. *β*-tubulin: 49.89 ± 9.13% versus 21.94 ± 6.72%, *P* < 0.01. Vimentin: 44.48 ± 12.48% versus 18.83 ± 2.25%, *P* < 0.01). There was no difference between the curcumin-treated group and the controls.

### 3.5. The Changes of the Mitochondrial Membrane Potential after SDT

**Figure 5:**
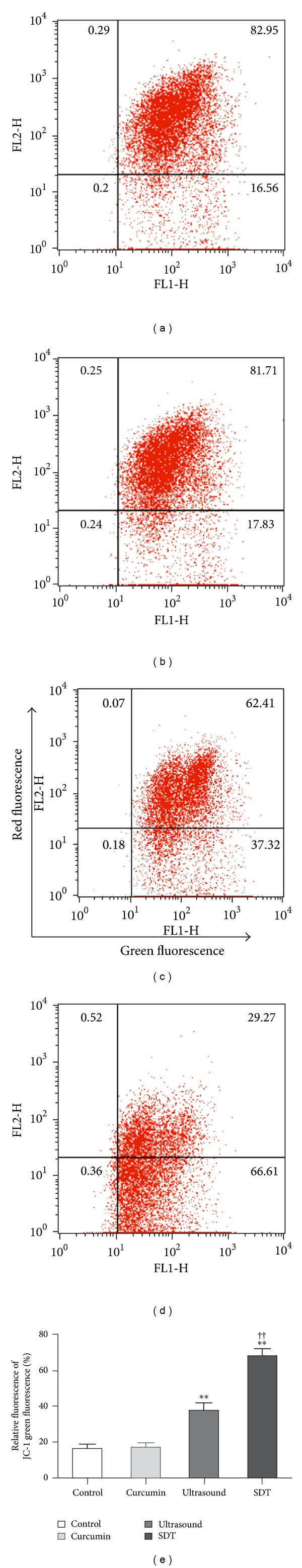
The change in mitochondrial membrane potential by curcumin SDT. (a) is control; (b) is curcumin alone; (c) is ultrasound irradiation alone; (d) is curcumin SDT; (e) is The representative dot plots from a single analysis are shown with the percentage of the cells in the lower right (LR) quadrant that emits only green fluorescence indicating the depolarized mitochondrial membrane. ^††^ *P* < 0.01 versus ultrasound alone; **P < 0.01 versus control.

As shown in Figure 5, the relative green signals of normal macrophages (Figure 5(a)) and cells with curcumin alone (Figure 5(b)) were 16.40 ± 2.44% and 17.14 ± 2.17% (versus control, *P* > 0.05), respectively. Those of the macrophages exposed to ultrasound alone (Figure 5(c)) and the macrophages subjected to curcumin SDT (Figure 5(d)) were 37.78 ± 4.17% (versus control, *P* < 0.01) and 68.23 ± 3.80% (versus control and ultrasound alone group, *P* < 0.01), respectively. There is a substantial shift in fluorescence emission, indicating a decrease in MMP in the SDT group (Figure 5(e)) compared to other groups (*P* < 0.01). 

## 4. Discussion

PDT is based on the principle of energy transfer from light to a photosensitizer to tissue. When an excited photosensitizer returns to the ground state and interacts with molecular oxygen, reactive oxygen species (ROS) are formed [22]. ROS promote photoinduced damage to biological molecules including lipids, proteins, and DNA [23]. Consequently, cell death occurs. The mechanism of the cytotoxicity in SDT seems to be theoretically similar to that in PDT. In SDT, the activation of HPDs through acoustic cavitation by ultrasound is attributed to the generation of active oxygen [24]. When a sonosensitizer is exposed to sonoluminescent light, it is activated from its ground state into an excited state; as the activated sonosensitizer returns to the ground state, the energy is released. Functional groups are required to accomplish this energy transition. The more conjugated macro-*π*  bonds inside sensitizer structures, the longer the absorption wavelength of the sensitizers. The number of macro-*π*  bonds inside curcumin is less than that of HPDs, so the absorption wavelength of curcumin is shorter than that of HPDs. Through measurement of the absorption spectrum, it was discovered that curcumin absorbed light at wavelength of less than 520 nm. The penetration depth of light depends on the wavelength. For example, wavelengths of 600–1000 nm can penetrate around 8–10 mm [25]. To the issue resolve penetration, ultrasound was used to activate curcumin in this study. 

During SDT, accumulation of the sensitizer in the target lesion is vital. The sensitizer targets and accumulates in metabolically active inflammatory cells, such as macrophages in an atheromatous plaque [2]. In this study, the uptake of curcumin by macrophages was detected. It was shown the accumulation of curcumin in macrophages increased in accordance with its concentration. The cytotoxicity curcumin also depended on its concentration. The results indicated that curcumin concentration over 81.4 **μ**mol/L would kill macrophages; on the contrary, curcumin concentrations below 13.6 **μ**mol/L did not exhibit intracellular drug fluorescence (data not shown). Liposoluble sensitizers likely enter cells through LDL-R [26], and because curcumin is liposoluble, it probably enters macrophages through this receptor. 

The cell survival rate in the curcumin SDT group was much lower than that in the ultrasound alone group under the same exposure conditions, while curcumin alone had little effect (Figure 2). Cell viability decreased gradually as the amount of ultrasound irradiation increased. This indicated that curcumin SDT can effectively kill macrophages in vitro. Moreover, cell viability in curcumin SDT was not altered when cells contained only intracellular curcumin. The MTT test provided some information concerning the function of mitochondria; however, it did not assess the late, irreversible changes that would indicate the mode of cell death. The Hoechst-PI assay was therefore more informative than the MTT assay. Apoptotic nuclei presented bright blue fluorescence spots accompanied by nucleus deformation while necrotic nuclei presented pink fluorescence (Figure 3(B4)). Because of resistance to fluorescence dye, the nuclei of live cells presented uniform blue fluorescence (Figure 3(B1)). In this study, ultrasound exposure alone could induce cell death, which became obvious when the amount of irradiation was augmented. Furthermore, this effect was highly enhanced when curcumin was added to the cells. The ratios of both apoptotic and necrotic cells increased. There was a synergistic relationship between curcumin and ultrasound. Therefore, curcumin may be a promising natural sonosensitizer when used at the proper concentration combined with the appropriate amount of ultrasound irradiation for treatment of atherosclerosis. Hematoporphyrin-SDT induced apoptosis of tumor cells through a mechanism that involved the mitochondria-caspase signaling pathway [27]. The mechanism of macrophage apoptosis induced by curcumin SDT may also involve activation of the mitochondria-caspase signaling pathway. 

Oxidative stress induced by PDT can affect several types of biomacromolecules including proteins, lipids, and DNA. Deleterious effects of PDT on the cytoskeletal proteins have been documented [28, 29]. SDT may also affect the cytoskeleton through a mechanism similar to that of PDT. Cytoskeletal F-actin might represent an important target for the SDT treatment [30]. In this study,  *α*-actin, *β*-tubulin, and vimentin were detected. The fluorescence signal of cytoskeletal proteins in the cells treated with ultrasound alone was partially attenuated, and this attenuation was greatly enhanced by adding curcumin. Cytoskeletal filaments were cleaved and formed clusters. The plasma membrane lost its normal structure and became deformed as blebs. No obvious deformation of the cytoskeleton was observed in cells treated with curcumin alone or controls. It is possible that the disruption of the cytoskeleton was one of the causes of cell death induced by curcumin SDT. 

Mitochondria-mediated cell death plays a crucial role in the pathophysiology of atherosclerosis. The loss of mitochondrial membrane potential was the upstream event for apoptosis [31]. Opening of the mitochondrial permeability transition pore (mPTP) induces swelling of mitochondria, leading to rupture of the mitochondrial outer membrane (MOM), and rupture of the MOM results in release of cytochrome c into the cytosol, triggering apoptosome formation [32]. The voltage-dependent anion channel (VDAC) lies in the outer mitochondrial membrane (OMM) and forms a common pathway for the exchange of metabolites between the mitochondria and the cytosol, thus playing a crucial role in the regulation of metabolic and energetic functions of mitochondria. VDAC appears to be a convergence point for a variety of cell survival and cell death signals, mediated by its association with various ligands and proteins [33, 34]. It was also proposed that VDAC and tubulin form a supercomplex with MtCK, which is structurally and functionally coupled to the ATP synthasome [35]. Actin-VDAC interactions are not a species-specific oddity and may be a more general phenomenon, the role of which ought to be further investigated [36]. Thus, VDAC interactions with actin and tubulin may have broader implications for various mitochondrial processes, including interactions between mitochondria and the cytoskeleton, in turn affecting mitochondrial dynamics [33]. In the present study, we have shown that ultrasound with or without curcumin results in the loss of mitochondrial membrane potential, and this loss was greatly enhanced by adding curcumin. The precise mechanism of how curcumin SDT linked to these mitochondrial events remains to be determined.

Currently, many possible sonosensitizers have been investigated, but few are approved for clinical use. Curcumin is widely used as a coloring and flavoring spice. Our results suggest that curcumin SDT may therefore be a useful clinical treatment for atherosclerosis. Whether curcumin SDT can induce atherosclerotic plague regression will require further study in animal models.

## 5. Conclusions

Curcumin had sonodynamic effect on THP-1-derived macrophages. Curcumin SDT decreased macrophages viability obviously and induced apoptosis or necrosis of macrophages. Both loss of mitochondrial membrane potential and morphological changes of cytoskeleton were apparent after treatment with curcumin SDT. In conclusion, curcumin is a new sonosensitizer, and curcumin SDT could be a promising treatment for atherosclerosis.

## References

[1] Gautier EL, Huby T, Witztum JL (2009). Macrophage apoptosis exerts divergent effects on atherogenesis as a function of lesion stage. *Circulation*.

[2] Estronca LM, Silva JC, Sampaio JL (2012). Molecular etiology of atherogenesis—in vitro induction of lipidosis in macrophages with a new LDL model. *PLoS One*.

[3] Kzhyshkowska J, Neyen C, Gordon S (2012). Role of macrophage scavenger receptors in atherosclerosis. *Immunobiology*.

[4] Waksman R, McEwan PE, Moore TI (2008). PhotoPoint photodynamic therapy promotes stabilization of atherosclerotic plaques and inhibits plaque progression. *Journal of the American College of Cardiology*.

[5] Lee DK, Choi Y, Shon SM (2011). Atorvastatin and clopidogrel interfere with photosensitization in vitro. *Photochemical and Photobiological Sciences*.

[6] Waksman R, Leitch IM, Roessler J (2006). Intracoronary photodynamic therapy reduces neointimal growth without suppressing re-endothelialisation in a porcine model. *Heart*.

[7] Ninomiya K, Ogino C, Oshima S (2012). Targeted sonodynamic therapy using protein-modified TiO2 nanoparticles. *Ultrasonics Sonochemistry*.

[8] Ohmura T, Fukushima T, Shibaguchi H (2011). Sonodynamic therapy with 5-aminolevulinic acid and focused ultrasound for deep-seated intracranial glioma in rat. *Anticancer Research*.

[9] El-Sikhry HE, Miller GG, Madiyalakan MR, Seubert JM (2011). Sonodynamic and photodynamic mechanisms of action of the novel hypocrellin sonosensitizer, SL017: mitochondrial cell death is attenuated by 11, 12-epoxyeicosatrienoic acid. *Investigational New Drugs*.

[10] Araújo CAC, Leon LL (2001). Biological activities of Curcuma longa L. *Memorias do Instituto Oswaldo Cruz*.

[11] Buhrmann C, Mobasheri A, Busch F (2011). Curcumin modulates nuclear factor kappaB (NF-kappaB)-mediated inflammation in human tenocytes in vitro: role of the phosphatidylinositol 3-kinase/Akt pathway. *The Journal of Biological Chemistry*.

[12] Samuhasaneeto S, Thong-Ngam D, Kulaputana O, Suyasunanont D, Klaikeaw N (2009). Curcumin decreased oxidative stress, inhibited NF-*κ*B activation, and improved liver pathology in ethanol-induced liver injury in rats. *Journal of Biomedicine and Biotechnology*.

[13] Dairaku I, Han Y, Yanaka N, Kato N (2010). Inhibitory effect of curcumin on IMP dehydrogenase, the target for anticancer and antiviral chemotherapy agents. *Bioscience, Biotechnology and Biochemistry*.

[14] Zingg JM, Hasan ST, Cowan D (2012). Regulatory effects of curcumin on lipid accumulation in monocytes/macrophages. *Journal of Cellular Biochemistry*.

[15] Quiles JL, Mesa MD, Ramírez-Tortosa CL (2002). Curcuma longa extract supplementation reduces oxidative stress and attenuates aortic fatty streak development in rabbits. *Arteriosclerosis, Thrombosis, and Vascular Biology*.

[16] Kawai Y (2011). Immunochemical detection of food-derived polyphenols in the aorta: macrophages as a major target underlying the anti-atherosclerotic activity of polyphenols. *Bioscience, Biotechnology and Biochemistry*.

[17] Wongcharoen W, Phrommintikul A (2009). The protective role of curcumin in cardiovascular diseases. *International Journal of Cardiology*.

[18] Park K, Lee JH (2007). Photosensitizer effect of curcumin on UVB-irradiated HaCaT cells through activation of caspase pathways. *Oncology Reports*.

[19] Osto E, Kouroedov A, Mocharla P (2008). Inhibition of protein kinase C*β* prevents foam cell formation by reducing scavenger receptor A expression in human macrophages. *Circulation*.

[20] Gao Q, Wang F, Guo S (2011). Sonodynamic effect of an anti-inflammatory agent—emodin on macrophages. *Ultrasound in Medicine and Biology*.

[21] Chen CC, Liou SW, Chen CC (2011). Coenzyme Q10 reduces ethanol-induced apoptosis in corneal fibroblasts. *PLoS ONE*.

[22] Fantini F, Greco A, Cesinaro AM (2008). Pathologic changes after photodynamic therapy for basal cell carcinoma and Bowen disease: a histologic and immunohistochemical investigation. *Archives of Dermatology*.

[23] Qiang YG, Yow CMN, Huang Z (2008). Combination of photodynamic therapy and lmmunomodulation: current status and future trends. *Medicinal Research Reviews*.

[24] Rosenthal I, Sostaric JZ, Riesz P (2004). Sonodynamic therapy—a review of the synergistic effects of drugs and ultrasound. *Ultrasonics Sonochemistry*.

[25] Mitra S, Foster TH (2004). Crabogen breathing significantly enhances the penetration of red light in murine tumours in vivo. *Physics in Medicine and Biology*.

[26] Kereiakes DJ, Szyniszewski AM, Wahr D (2003). Phase I drug and light dose-escalation trial of motexafin lutetium and far red light activation (phototherapy) in subjects with coronary artery disease undergoing percutaneous coronary intervention and stent deployment: procedural and long-term results. *Circulation*.

[27] Tang W, Liu Q, Zhang J, Cao B, Zhao P, Qin X (2010). In vitro activation of mitochondria-caspase signaling pathway in sonodynamic therapy-induced apoptosis in sarcoma 180 cells. *Ultrasonics*.

[28] Vantieghem A, Xu Y, Assefa Z (2002). Phosphorylation of Bcl-2 in G2/M phase-arrested cells following photodynamic therapy with hypericin involves a CDK1-mediated signal and delays the onset of apoptosis. *The Journal of Biological Chemistry*.

[29] Uzdensky A, Kolpakova E, Juzeniene A, Juzenas P, Moan J (2005). The effect of sub-lethal ALA-PDT on the cytoskeleton and adhesion of cultured human cancer cells. *Biochimica et Biophysica Acta*.

[30] Zhao X, Liu Q, Tang W (2009). Damage effects of protoporphyrin IX—sonodynamic therapy on the cytoskeletal F-actin of Ehrlich ascites carcinoma cells. *Ultrasonics Sonochemistry*.

[31] Koppikar SJ, Choudhari AS, Suryavanshi SA, Kumari S, Chattopadhyay S, Kaul-Ghanekar R (2010). Aqueous cinnamon extract (ACE-c) from the bark of Cinnamomum cassia causes apoptosis in human cervical cancer cell line (SiHa) through loss of mitochondrial membrane potential. *BMC Cancer*.

[32] Miura T, Tanno M (2011). The mPTP and its regulatory proteins: final common targets of signalling pathways for protection against necrosis. *Cardiovascular Research*.

[33] Shoshan-Barmatz V, Ben-Hail D (2012). VDAC, a multi-functional mitochondrial protein as a pharmacological target. *Mitochondrion*.

[34] Guzun R, Gonzalez-Granillo M, Karu-Varikmaa M (2012). Regulation of respiration in muscle cells in vivo by VDAC through interaction with the cytoskeleton and MtCK within Mitochondrial Interactosome. *Biochimica Biophysica Acta*.

[35] Saks V, Guzun R, Timohhina N (2010). Structure-function relationships in feedback regulation of energy fluxes in vivo in health and disease: mitochondrial Interactosome. *Biochimica et Biophysica Acta*.

[36] Roman I, Figys J, Steurs G, Zizi M (2006). Direct measurement of VDAC-actin interaction by surface plasmon resonance. *Biochimica et Biophysica Acta*.

